# TRIM68 Negatively Regulates IFN-β Production by Degrading TRK Fused Gene, a Novel Driver of IFN-β Downstream of Anti-Viral Detection Systems

**DOI:** 10.1371/journal.pone.0101503

**Published:** 2014-07-07

**Authors:** Claire Wynne, Elisa Lazzari, Siobhán Smith, Eoghan M. McCarthy, Joan Ní Gabhann, Lara E. Kallal, Rowan Higgs, Sally Ann Cryan, Christine A. Biron, Caroline A. Jefferies

**Affiliations:** 1 Molecular and Cellular Therapeutics, Royal College of Surgeons in Ireland, Dublin, Ireland; 2 School of Biological Sciences, Dublin Institute of Technology, Dublin, Ireland; 3 Department of Molecular Microbiology and Immunology, Brown University, Providence, Rhode Island, United States of America; 4 School of Pharmacy, Royal College of Surgeons in Ireland, Dublin, Ireland; 5 Trinity Centre for Bioengineering, Trinity College Dublin, Dublin, Ireland; University of Tennessee Health Science Center, United States of America

## Abstract

In recent years members of the tripartite motif-containing (TRIM) family of E3 ubiquitin ligases have been shown to both positively and negatively regulate viral defence and as such are emerging as compelling targets for modulating the anti-viral immune response. In this study we identify TRIM68, a close homologue of TRIM21, as a novel regulator of Toll-like receptor (TLR)- and RIG-I-like receptor (RLR)-driven type I IFN production. Proteomic analysis of TRIM68-containing complexes identified TRK-fused gene (TFG) as a potential TRIM68 target. Overexpression of TRIM68 and TFG confirmed their ability to associate, with TLR3 stimulation appearing to enhance the interaction. TFG is a known activator of NF-κB via its ability to interact with inhibitor of NF-κB kinase subunit gamma (IKK-γ) and TRAF family member-associated NF-κB activator (TANK). Our data identifies a novel role for TFG as a positive regulator of type I IFN production and suggests that TRIM68 targets TFG for lysosomal degradation, thus turning off TFG-mediated IFN-β production. Knockdown of TRIM68 in primary human monocytes resulted in enhanced levels of type I IFN and TFG following poly(I:C) treatment. Thus TRIM68 targets TFG, a novel regulator of IFN production, and in doing so turns off and limits type I IFN production in response to anti-viral detection systems.

## Introduction

Innate immune receptors play key roles in viral recognition and activation of transcription factors important for driving both type I IFN and pro-inflammatory cytokine production. Production of type I IFN (IFN-α and IFN-β) following viral and bacterial infection is a critical step in the innate immune response. Whilst important for both anti-viral and anti-bacterial immunity, IFNs and pro-inflammatory cytokines can become pathogenic when overproduced, resulting in inflammatory autoimmune diseases such as systemic lupus erythematosus (SLE) or Crohn's disease. Thus, proteins that function to turn off and limit the production of such cytokines are important immunoregulatory factors. Members of the tripartite motif-containing (TRIM) family of E3 ubiquitin ligases have been shown to both positively [1]–[6] and negatively [7]–[13] regulate immune responses, mainly by ubiquitinating key signalling intermediates and thus either enhancing their activity or targeting them for ubiquitin-mediated degradation, respectively.

Viral recognition, broadly speaking, results in activation of pathways regulating the activity of either NF-κB or interferon regulatory factor (IRF) 3 or IRF7. Anti-viral sensors principally detect viral nucleic acid, and under pathogenic circumstances can detect RNA and DNA released from damaged host cells. They include the Toll-like receptors (TLRs), TLR3, 7 and 9, which are endosomally located and the cytosolic RNA-sensing RIG-like helicase receptors (RLRs). RIG-I and melanoma differentiation-associated protein 5 (MDA-5) have been shown to recognise viral RNA whereas multiple DNA-receptors exist (reviewed in [14], [15]). Once activated these PRRs recruit adaptor proteins, such as TIR domain-containing adaptor protein inducing interferon-β (TRIF) and myeloid differentiation primary response gene 88 (MyD88) to the TLRs and mitochondrial anti-viral signalling protein (MAVS) to the RLRs, which facilitate the formation of signalling complexes that result ultimately in the activation of downstream kinases such as IκB kinases and TANK-binding kinase 1 (TBK1). Together these regulate the activity of the transcription factors NF-κB and the IRF family members (IRF3 and 7) and the subsequent production of pro-inflammatory cytokines and type I IFNs (reviewed in [16]).

Recently a role for TRIMs in anti-viral immunity has been highlighted (reviewed in [17]). TRIM proteins act as either positive or negative regulators of type I IFN production, utilising their E3 ligase activity for activation (via K63-linked polyubiquitination) or degradation (via K48-linked polyubiquitination) of key signalling molecules on viral recognition pathways. Structurally, the TRIM protein family are characterised by the presence of a RING finger domain, one or two B-box domains and a coiled-coil domain in their N-terminal region [18]. The most common C-terminal domain expressed by TRIMs is the SPRY domain (also known as a B30.2 domain), a domain known to regulate anti-viral immune responses [19]. TRIM21 was first described as a target for autoantibody production in SLE and Sjögren's syndrome (SS) [20]–[22] and was amongst the first of the TRIM proteins shown to negatively regulate IFN production [7], [23]. As a negative regulator, TRIM21 targets the IRF family members IRF3 and IRF7 for degradation [7], [8]. However, a positive role for TRIM21 in driving pro-inflammatory cytokine production has also been demonstrated, underlining the complex role this protein plays in innate immune responses [24]–[26]. TRIM68 is most structurally and phylogenetically similar to TRIM21, with both TRIMs expressing a PRY/SPRY domain in their C-terminal region [27]. Little is known regarding a role for TRIM68 in regulating innate immune signalling, however it has been shown to be significantly upregulated in prostate cancer cells [28], [29] and has also been shown to cross react with melanoma inhibitor of apoptosis protein (ML-IAP)-specific cytotoxic T cells isolated from melanoma patients [30]. Although TRIM68 has been identified as a cellular target for autoantibody responses in SS and SLE [31], whether it has any role in immune responses remain unknown.

Our findings indicate a hitherto unsuspected role for TRIM68 as a negative regulator of type I IFN production. In this respect, we identify TRK-fused gene (TFG) as TRIM68's target, a protein known to interact with the scaffold proteins inhibitor of NF-κB kinase subunit gamma (IKK-γ) and TRAF family member-associated NF-κB activator (TANK), thus positively facilitating NF-κB activation and pro-inflammatory cytokine production [32]. In this study we identify a positive role for TFG in driving IFN-β production, with knockdown of TFG significantly inhibiting TRIF-driven IFN-β induction. We demonstrate that TRIM68 binds and polyubiquitinates TFG, leading to its lysosomal degradation and hence inhibition of TFG-dependent signalling. We show that TRIM68 knockdown in human monocytes results in an increase in IFN-β and TFG levels post TLR3 stimulation, further validating the negative role TRIM68 exerts on these responses. This is the first report that demonstrates a role for TFG as a positive regulator of type I IFN production and a role for TRIM68 as a negative regulator of this process.

## Materials and Methods

### Ethics Statement

In the case of human blood collection for monocyte isolation, participants provided their written informed consent to participate in this study. All samples were collected with informed consent and used in strict accordance with ethical approval from Royal College of Surgeons in Ireland research ethics committee REC269.

### Cell culture

Human embryonic kidney (HEK) 293T cells (Unitech Ltd) and HeLa cells (ATCC) were cultured in DMEM medium (Biosera) supplemented with 10% FCS (Biosera), penicillin and streptomycin (100 µg/ml) (Biosera). Human PBMCs were isolated from whole blood by density gradient centrifugation using Ficoll-Paque Plus (GE Healthcare). Human monocytes were subsequently extracted from PBMCs by positive selection using CD14 beads (Miltenyi Biotec) and cultured in RPMI-1640 medium (Biosera) supplemented with 10% FCS and penicillin–streptomycin (100 µg/ml). Following stimulation, supernatants were collected and RANTES levels determined by ELISA (R&D Systems) according to the manufacturer's recommendations.

### Plasmids and reagents

FLAG-TRIM68 was a kind gift from Dr. Shigetsugu Hatakeyama, (Hokkaido University Graduate School of Medicine, Sapporo, Japan). FLAG-TRIF, FLAG-TBK1, FLAG-IRF3, FLAG-RIG-I, FLAG-MAVS and the IFN-β luciferase reporter construct were kind gifts from Prof. Katherine Fitzgerald (University of Massachusetts Medical School, Worcester, MA, USA). The κB-luciferase reporter construct was a kind gift from Dr. R Hofmeister (University of Regensburg, Germany). MYC-MyD88 construct was a kind gift from Dr. Aisling Dunne (Trinity College Dublin, Ireland). HA-Ubiquitin was a kind gift from Dr. Andrew Bowie (Trinity College Dublin, Ireland). GFP-TRIM68 construct was a kind gift from Dr. David Rhodes (Cambridge Institute for Medical Research, UK). MYC-TFG was a kind gift from Dr. Angela Greco (The Foundation of the Carlo Besta Neurological Institute, Italy). Ligands used were 5′ppp-dsRNA and polyinosinic:polycytidylic acid (poly(I:C)) (Invivogen). shRNAs used were human TRIM68 shRNA and human TFG shRNA (Sigma). Primary antibodies used were anti-alpha actinin, anti-HA (Santa Cruz Biotechnologies), anti-FLAG (Sigma), anti-TFG and anti-MYC (Abcam).

### Luciferase reporter gene assays

HEK293T or HeLa cells were seeded at 2×10^5^/ml and transiently transfected for 18–24 hr with 40 ng of the indicated reporter constructs, increasing amounts of a TRIM68/TFG construct (10 ng, 50 ng and 100 ng) and an appropriate driver construct (50 ng). In the case of TFG shRNA work, HEK293T cells were transiently transfected with 40 ng of p125 and 250 ng of shRNA (Scrambled or TFG) and 24 hr later with 50 ng of TRIF. All transfections were performed using Metafectene (Biontex) according to the manufacturer's recommendations. Luciferase activity was standardised to renilla luciferase plasmid activity to normalise for transfection efficiency.

### Confocal microscopy

HeLa cells were transfected with 2 µg of GFP-TRIM68 for 18–24 hr and were then treated with poly(I:C) (20 µg/ml) for 6 or 24 hr. Cells were fixed with methanol/acetone (Sigma) at a 1∶1 ratio, stained with an anti-TFG antibody and mounted in DAPI containing mounting media (Dako). Localisation of TFG was visualised using an Alexa fluor 546 conjugated anti-rabbit secondary antibody (Molecular Probes) and cells were imaged by confocal microscopy on a Zeiss LSM 510 META (Oberkochen, Germany).

### Western blot and immunoprecipitation analysis

For immunoprecipitation experiments, HEK293T cells were seeded at 5×10^5^/ml, in 10 ml, in 10 cm dishes and transfected with a total of 4 µg of plasmid for 18–24 hr. Cells were lysed on ice in radioimmunoprecipitation lysis buffer (1×PBS, 1% Nonidet P-40, 0.5% Na-deoxycholate, 0.1% SDS, 1 mM KF, 1 mM Na_3_VO_4_, 10 µg/ml leupeptin, and 1 mM PMSF) followed by immunoprecipitation with either anti-FLAG (M2), anti-TFG or anti-HA (Y-11/12CA5) pre-coupled to protein-G Sepharose beads (Sigma). In some cases HEK293T cells were treated with the lysosomal inhibitor NH_4_Cl (20 mM) for 18 hr as indicated or with the proteosomal inhibitor MG132 (10 µM) (Sigma) for 2 hr.

### Trypsin digest and MS/MS analysis

10% acrylamide gels were stained using Pierce Silver Staining Kit (Thermo Scientific) as per manufacturers' instructions. Gel pieces were excised, destained and digested in sequencing grade modified trypsin (Promega) (20 ng/µl). The resulting peptide mixtures were resuspended in 0.1% formic acid and analysed by nanoelectrospray liquid chromatography mass spectrometry (Nano-LC MS/MS). Spectra were searched using the SEQUEST algorithm against the International Protein Index (IPI) database (http://www.ebi.ac.uk/IPI/IPIhelp.html).

### Real-time PCR

Total RNA was extracted from cell cultures using an RNeasy kit (Qiagen) and reverse transcribed to cDNA using Omniscript reverse transcriptase (Qiagen) according to manufacturer's recommendations. Quantitative real-time PCR was performed using SYBR Green Taq ReadyMix (Sigma) on an Applied Biosystems 7500 real-time PCR machine and the data was normalised to an *18S RNA* reference. *IFN-β*, *TRIM68* and *18S RNA* templates were amplified using primers (sequences available on request) with an annealing temperature of 56°C. Real-time PCR data was analysed using the 2^−ΔΔCt^ method [33].

### Cloning of TRIM68 PRY/SPRY

Briefly, the 780 bp TRIM68 PRY/SPRY region was amplified by PCR from genomic DNA. The primers used were as follows: foward 5′ atgttgcaggatattcag -3′ and reverse 5′ – gtcctccccatccaggga -3′. The foward and reverse primers created Not1 and HindIII sites, respectively, and the PCR products were ligated into these sites in the pCDNA3.1/myc-His(-) vector. Transformants were validated by sequencing (MWG Eurofins Genetics, Ebersberg, Germany).

### Microparticle manufacture and TRIM68 shRNA knockdown

Poly (lactic-co-glycolic acid) (PLGA) 503 (Boehringer Ingelheim) microparticles containing either scrambled (Scr) or TRIM68 shRNA (Sigma) were prepared using a double emulsion solvent evaporation method. Lyophilised microparticles were sized using a Malvern Mastersizer 2000 (Malvern Instruments) and the encapsulation efficiency determined using a Quant-iT PicoGreen assay (Invitrogen). Fluorescence was detected at 485 nm excitation and 535 nm emission wavelengths using the Victor Wallac Multiplate Reader. Human monocytes were plated at 2×10^6^/ml and treated with microparticles containing 150 ng of shRNA (Scr or TRIM68) dissolved in 150 µl of serum free RPMI-1640 medium for 48 hr. 24 hr post microparticle addition, cells were treated with poly(I:C) (20 µg/ml) for the remaining 24 hr before real-time PCR analysis or western blotting was performed.

### Statistical analysis

All data are presented as the average of three separate experiments ± standard error of the mean (S.E.M). Statistical significance for reporter gene assays was determined using a two-way ANOVA analysis followed by a Bonferroni post test in which everything was compared back to the 0 ng data set. For all other comparisons, the two-tailed Student t-test was used. Data were deemed to be significantly different at p value <0.05 (*) (**p<0.01, ***p<0.001). GraphPad Prism 5.0 software was used to test for statistical significance.

## Results

### TRIM68 is a novel negative regulator of TLR/RLR-driven IFN-β promoter activity

**Figure 1:**
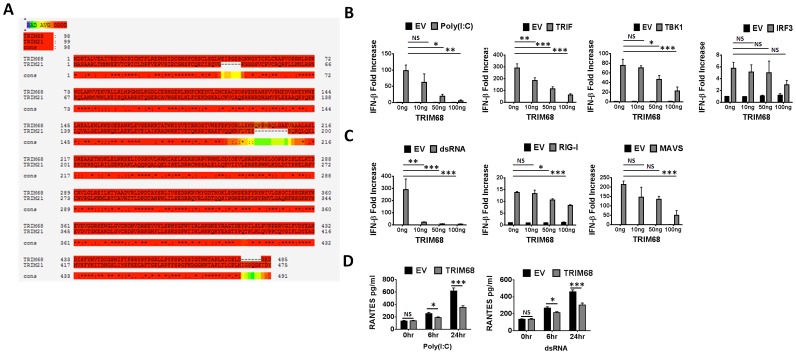
TRIM68 is structurally similar to TRIM21 and down-regulates type I IFN production downstream of TBK1. (A) Human TRIM68 and TRIM21 amino acid sequence alignment using T-Coffee (http://tcoffee.crg.cat/apps/tcoffee/do:regular). An * (asterisk) indicates positions which have a single, fully conserved residue. A: (colon) indicates conservation between groups of strongly similar properties - scoring >0.5 in the Gonnet PAM 250 matrix. A. (period) indicates conservation between groups of weakly similar properties - scoring <0.5 in the Gonnet PAM 250 matrix. (B–C) HeLa cells were transfected with 40 ng full-length IFN-β promoter, empty vector (EV) control and increasing amounts of TRIM68 as indicated for 18 hr and were then stimulated with 20 µg/ml poly(I:C) for 24 hr. In all other cases HEK293T cells were transfected with 40 ng full-length IFN-β promoter and were co-transfected with 50 ng of the relevant promoter drivers TRIF, TBK1, IRF3 (B), or dsRNA, RIG-I, MAVS (C) as well as with empty vector (EV) control and increasing amounts of TRIM68 as indicated. Cells were assayed for relative fold increase of reporter gene activity 18–24 hr post-transfection. (D) HeLa cells were transiently transfected with 1 µg of TRIM68 together with 1 µg/ml 5′ppp-dsRNA for 6 and 24 hr or 18 hr later with 20 µg/ml poly(I:C) for 6 and 24 hr before supernatants were collected for RANTES ELISA. In all cases presented data is graphed from the average of three separate experiments ± S.E.M. *p<0.05 was considered significant.

Both TRIM21 and TRIM68 have previously been identified as targets for autoantibody production in SLE [34], [35]. In addition to this, bioinformatic analysis indicates that TRIM68 is the most closely related TRIM family member to TRIM21, with both TRIMs clustering together in 66% of replicate trees [27]. *TRIM21* and *TRIM68* are also located in close proximity to each other within a cluster of TRIMs on chromosome 11 at positions p15.4 and p15.5, respectively (27). Like TRIM21 and the other proteins found in this cluster (with the exception of TRIM3), TRIM68 contains a SPRY/PRY domain, a domain which has been shown to be important in immune regulation (reviewed in [36]). T-Coffee alignment of the amino acid sequence of both TRIM members generated a sequence similarity score of 98% (Figure 1A), whereas a Clustal W alignment indicates that TRIM68 and TRIM21 share a total of 43.16% sequence identity (Figure S1), suggesting that TRIM68 may have split from TRIM21 *via* gene duplication. To determine whether TRIM68 also functions as a negative regulator of type I IFN responses similar to TRIM21, we assessed its ability to affect IFN-β promoter activity. The addition of increasing amounts of a plasmid expressing TRIM68 significantly inhibited poly(I:C)-, TRIF- and TBK1-driven IFN-β promoter activity but had no effect on IRF3-driven IFN-β promoter activity (Figure 1B). To determine the potential role of TRIM68 on RIG-I-driven IFN-β promoter activity, cells were transiently transfected with RIG-I ligand dsRNA, RIG-I and its adaptor protein MAVS, and increasing amounts of TRIM68 (Figure 1C). As with TRIF-driven IFN-β promoter activity, TRIM68 negatively regulated dsRNA, RIG-I- and MAVS-driven reporter gene activation (Figure 1C). RANTES levels were also significantly decreased in cells in which TRIM68 was overexpressed post poly(I:C) and dsRNA treatment, suggesting TRIM68 negatively regulates TLR3- and RLR-driven RANTES release (Figure 1D). Collectively our results indicate a novel negative regulatory role for TRIM68 downstream of both TLR- and RLR-driven IFN-β production and suggest the target for TRIM68 lies downstream of TBK1 but upstream of IRF3. Interestingly TRIM68 also negatively regulated NF-κB promoter activity downstream of MyD88, TRIF and TBK1 suggesting that it works at a point of crosstalk between pathways controlling both type IFN production and NF-κB activation or alternatively suggesting that TRIM68 may have two different targets on IRF versus NF-κB pathways (Figure S2).

### TRIM68 interacts with TRK fused gene (TFG), a novel regulator of IFN-β production

**Figure 2:**
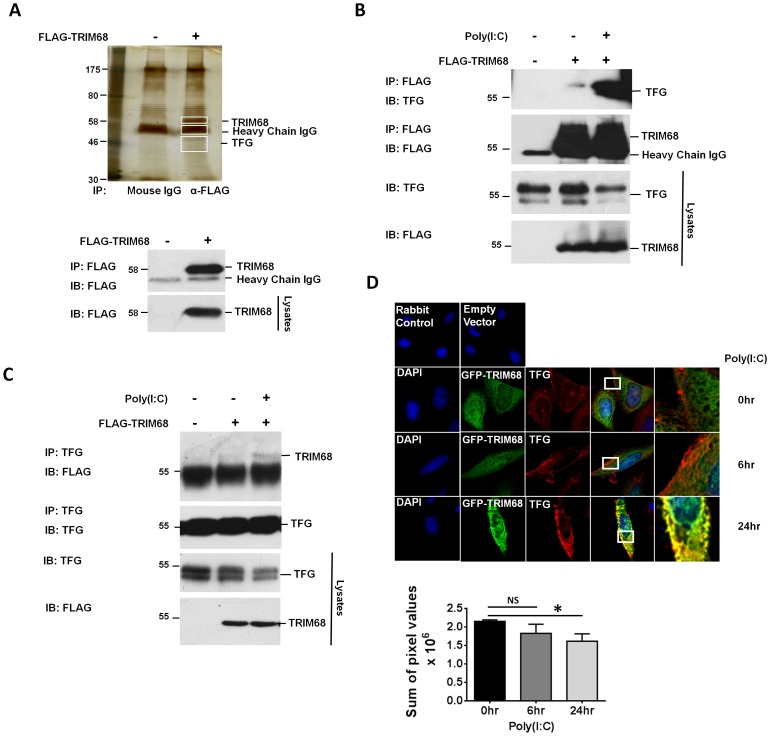
TRIM68 interaction with TRK-fused gene protein (TFG) increases post TLR3 stimulation. (A) TRIM68 bound proteins were immunoprecipitated from lysates with anti-FLAG coated agarose beads (Sigma). Immunoprecipitation using normal mouse IgG functioned as a negative control. Selected bands were excised, trypsin digested and analysed by mass spectrometry. A western blot showing corresponding lysates from the silver stained gel was immunoblotted for FLAG to verify successful transfection and immunoprecipitation of FLAG-TRIM68. Immunoblots shown are from a single experiment and are representative of two independent experiments. (B) HEK293T cells were transfected with 2 µg of EV and FLAG-TRIM68. TRIM68 containing complexes were immunoprecipitated using anti-FLAG coated beads and blotted for TFG and FLAG. Lysates were immunoblotted for TFG and FLAG. Immunoblots shown are from a single experiment and are representative of three independent experiments. (C) HEK293T cells were transfected with 2 µg of EV and FLAG-TRIM68. TFG containing complexes were immunoprecipitated using anti-TFG coated beads and immunoblotted for FLAG and TFG. Lysates were immunoblotted for TFG and FLAG. Immunoblots shown are from a single experiment and are representative of three independent experiments. (D) HeLa cells were transfected with 2 µg of GFP-TRIM68 (green) expression plasmid and stimulated with 20 µg/ml of poly(I:C) for 6 and 24 hr. Cells were fixed and then stained with anti-TFG before mounting in DAPI in order to visualise nuclei (blue). Images were taken under oil immersion at 63X magnification. Panels on the right contain zoomed images of white box in previous panel. Images shown are from a single experiment and are representative of three independent experiments. The raw integrated density value (sum of pixel values) was calculated using ImageJ software (1.48 4-bit Java) for TFG only images for each poly(I:C) time point. Presented data is graphed from the average of sixteen separate images ± S.E.M. *p<0.05 was considered significant.

Having demonstrated that TRIM68 inhibited both TLR- and RLR-driven type I IFN production, we next sought to identify potential targets for TRIM68. To do this, FLAG-TRIM68-containing immunocomplexes were separated by SDS PAGE, silver-stained and bands which were present in the FLAG-TRIM68 immunoprecipitated lane but not in the IgG control lane were excised and analysed by Nano-LC MS/MS (Figure 2A, Figure S3). TFG was identified as a potential target of TRIM68 and the ability of the two proteins to interact confirmed following overexpression of FLAG-tagged TRIM68 in HEK293T cells followed by western blotting of TRIM68-containing complexes for TFG (Figure 2B). The interaction between TRIM68 and TFG was greatly enhanced following 24 hr poly(I:C) stimulation, with a concomitant decrease in TFG levels also evident (Figure 2B). In order to further validate the interaction between TRIM68 and TFG, the immunoprecipitation was also carried out in reverse in which TFG-containing complexes were immunoblotted for TRIM68 with a resulting poly(I:C) inducible interaction between TRIM68 and TFG again being evident (Figure 2C). Finally, the interaction between TRIM68 and TFG was further confirmed by confocal microscopy, with transiently transfected GFP-tagged TRIM68 observed to interact with TFG following poly(I:C) treatment of HeLa cells for 24 hr (Figure 2D). Although in this image TFG appears to be stabilized post poly(I:C) treatment, quantification was carried out on multiple images using “ImageJ” software demonstrating that absolute TFG levels do in fact decrease compared with unstimulated controls, thus supporting the observation that TFG is degraded following poly(I:C) stimulation (Figure 2D, lower figure).

### TRIM68 polyubiquitinates TFG leading to its degradation via its RING domain which also plays an important role in TRIM68 negative inhibition on IFN-β production

**Figure 3:**
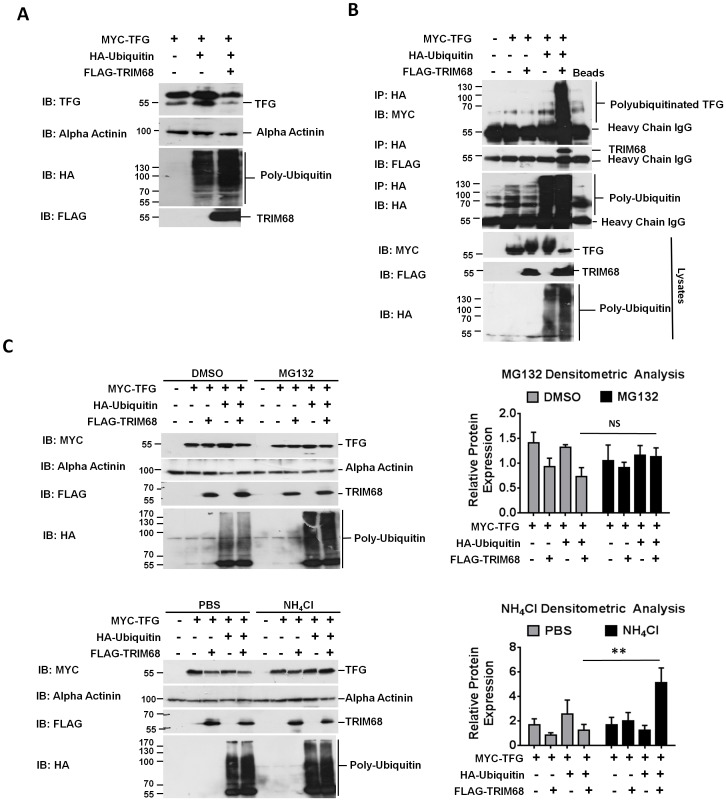
TRIM68 negatively regulates type I IFN production via polyubiquitination and lysosomal degradation of TFG. (A) HEK293T cells were co-transfected with MYC-TFG, HA-Ubiquitin, FLAG-TRIM68 and EV. Lysates were immunoblotted for TFG, alpha actinin, HA and FLAG. Immunoblots shown are from a single experiment and are representative of three independent experiments. (B) HEK293T cells were transfected with 1 µg of EV, MYC-TFG, HA-Ubiquitin and FLAG-TRIM68. Ubiquitin containing complexes were immunoprecipitated using anti-HA coated beads and immunoblotted for MYC, FLAG and HA. Lysates were immunoblotted for FLAG and HA. Immunoblots shown are from a single experiment and are representative of three independent experiments. (C) Cells were treated with proteasomal inhibitor MG132 (10 µM) and DMSO vehicle control for 2 hr or lysosomal inhibitor NH_4_Cl (20 mM) or PBS vehicle control for 18 hr before cells were lysed and immunblotted for MYC, alpha actinin, FLAG and HA. In all cases the immunoblots shown are from a single experiment and are representative of three independent experiments. Densitometry data is graphed from the average of three separate experiments ± S.E.M. of TFG normalised to alpha actinin.

Interestingly, we consistently observed reduced levels of TFG in cell lysates in the presence of TRIM68 following stimulation suggesting that TRIM68 may destabilise TFG. In keeping with this, we observed a marked decrease in TFG protein levels in cells co-expressing Ubiquitin, TFG, and TRIM68 even in the absence of stimulation, indicating that TFG may be ubiquitinated and hence targeted for degradation by TRIM68 (Figure 3A). In line with this hypothesis, MYC-tagged TFG was shown to be polyubiquitinated when co-expressed with both HA-tagged ubiquitin and FLAG-tagged TRIM68 (Figure 3B). Analysis of the nature of the polyubiquitin chains added on by TRIM68 proved to be inconclusive, suggesting that the TRIM68-mediated polyubiquitination of TFG process is not entirely dependent on either K48- or K63-linked ubiquitin chains and may involve mixed K48/K63-linked chains or chains using alternative linkages such as K29 (Figure S4). As ubiquitination can signal degradation of target proteins via either proteasomal or lysosomal pathways (reviewed in (37)), HEK293T cells overexpressing TFG, TRIM68 and Ubiquitin were treated with either the proteasome inhibitor, MG132 (or DMSO vehicle control) or the lysosomal degradation inhibitor, NH_4_Cl (or PBS vehicle control) and the effect of TRIM68 on TFG levels assessed by western blot analysis (Figure 3C). Whilst proteosomal inhibition had no effect on TFG degradation (Figure 3C, top panels), inhibition of lysosome-phagosome fusion by NH_4_Cl resulted in a significant accumulation of TFG in the presence of TRIM68 and Ubiquitin (Figure 3C, bottom panels), suggesting that TFG is in fact being ubiquitinated and degraded by TRIM68 *via* lysosomal degradative pathways.

### TFG, a novel driver of type I IFN production, increases in the absence of TRIM68

**Figure 4:**
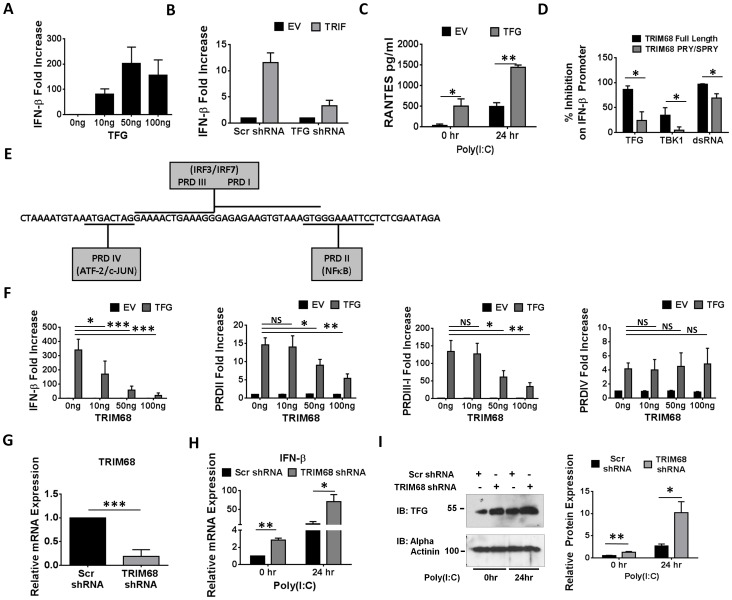
TRIM68 knockdown results in an increase in IFN-β and TFG levels post TLR3 stimulation. (A) HEK293T cells were transfected with 40 ng full-length IFN-β promoter and increasing amounts of TFG as indicated. Cells were assayed for relative fold increase of reporter gene activity 18–24 hr post-transfection. (B) HEK293T cells were transfected with 400 ng full-length IFN-β promoter and were co-transfected with 250 ng of Scrambled (Scr) or TFG shRNA as indicated and 24 hr later 50 ng of TRIF. Cells were assayed for relative fold increase of reporter gene activity 18–24 hr post-transfection. Presented data is graphed from one experiment. (C) HeLa cells were transiently transfected with 1 µg of TFG and 18 hr later were stimulated with 20 µg/ml poly(I:C) for 24 hr before supernatants were collected for RANTES ELISA. (D) HEK293T cells were transfected with 40 ng full-length IFN-β promoter and were co-transfected with 50 ng of TFG, TBK1 or dsRNA as well as with empty vector (EV) control and 50 ng of full length TRIM68 or TRIM68 mutant lacking the RING and B-box domain (TRIM PRY/SPRY) as indicated. Cells were assayed for relative fold increase of reporter gene activity 18–24 hr post-transfection and % inhibition on promoter activity was calculated. Presented data is graphed from the average of three separate experiments ± S.E.M. *p<0.05 was considered significant. (E) A schematic of the IFN-β promoter which consists of three principle transcription factor binding sites, the PRD IV region which binds the transcription factor ATF-2/c-Jun, the PRD III–I region which binds the transcription factors IRF3 and IRF7 and the PRD II region which binds NF-κB. (F) HEK293T cells were transfected with 40 ng full-length IFN-β promoter or the PRD segments (PRD II, PRD III-I, PRD IV) and 50 ng of TFG as well as with EV control and increasing amounts of TRIM68 as indicated. Cells were assayed for relative fold increase of reporter gene activity 18–24 hr post-transfection. (G–I) Human monocytes were treated with PLGA microparticles containing either Scrambled (Scr) or TRIM68 shRNA for 48 hr. Real-time PCR was used to measure TRIM68 mRNA levels post treatment (G). After treatment with PLGA microparticles, monocytes were stimulated with poly(I:C) for 24 hr before IFN-β mRNA was measured (H). Human monocytes which have been treated with shRNA containing microparticles as above were immunoblotted for TFG and alpha actinin. Immunoblots shown are from a single experiment and are representative of three independent experiments (I). In all other cases, presented data is graphed from the average of three separate experiments ± S.E.M. *p<0.05 was considered significant.

Given previous reports that TFG can positively influence pro-inflammatory cytokine production, we next set out to elucidate if TFG had any effect on type I IFN production. Transient transfection of HEK293T cells with increasing amounts of TFG lead to IFN-β promoter activation (Figure 4A). In addition, TFG knockdown in the same cells resulted in decreased TRIF-driven IFN-β promoter activity, indicating that TFG is indeed a positive regulator of IFN-β production in this system (Figure 4B). In contrast, overexpression of TFG in HeLa cells resulted in a significant increase in RANTES release both before and 24 hr after poly(I:C) stimulation further suggesting TFG functions as a positive regulator of the type I IFN response (Figure 4C). Having shown that TFG positively influences IFN-β production and TRIM68 promotes ubiquitination and degradation of TFG, we next set out to determine whether this ubiquitination was involved in the inhibition of IFN-β signalling. The activity of most E3 ligases, like TRIM68, is specified by a RING (Really Interesting New Gene) Finger domain. In order to investigate whether this domain played a role in how TRIM68 negatively regulated TFG-, TBK1- and dsRNA-driven IFN-β promoter activity, a mutant TRIM68 plasmid was cloned lacking the RING domain (TRIM68 PRY/SPRY) and cells were transiently transfected with TFG, TBK1 or dsRNA and increasing amounts of the mutant TRIM68 plasmid and % inhibition on IFN-β promoter activity was compared with full length TRIM68 (Figure 4D). The removal of the RING domain significantly decreased TFG-, TBK1- and dsRNA-driven inhibition on IFN-β promoter activity suggesting that this domain plays an important role in how TRIM68 exerts its negative effect on type I IFN production. The remaining inhibition exhibited on IFN-β promoter in the absence of the RING domain suggests that not all TRIM68 effects are RING domain-dependent. The IFN-β promoter has three principle transcription factor binding sites; the PRD IV region which binds the transcription factor ATF-2/c-Jun, the PRD III–I region which binds the transcription factors IRF3 and IRF7 and the PRD II region which binds NF-κB (Figure 4E). In order to investigate which binding sites on the IFN-β promoter TRIM68 could negatively regulate, we carried out TFG-driven reporter gene assays on the full length IFN-β promoter as well as each of the three segments (Figure 4E). In keeping with TFG being a target for TRIM68, the addition of increasing concentrations of a plasmid expressing TRIM68 significantly inhibited TFG-driven full length IFN-β promoter activity as well as TFG-driven PRD II and PRD III-I promoter activity whilst having no effect on TFG-driven PRD IV promoter activity (Figure 4F). Having shown that TRIM68 targets TFG for degradation *in vitro*, we next assessed the effects of TRIM68 knockdown on both IFN-β and TFG levels. Human monocytes were transfected with either scrambled (Scr) control shRNA or shRNA targeted against TRIM68 and knockdown efficiency determined by assessing TRIM68 mRNA levels (Figure 4G). A significant increase in both IFN-β mRNA levels and TFG protein levels before and after 24 hr poly(I:C) stimulation was evident in TRIM68 deficient monocytes (Figure 4H and 4I) with densitometric analysis shown on the right) consistent with TRIM68 negatively regulating type I IFN production *via* degradation of TFG. Thus, TRIM68, via inducing the degradation of TFG targets both NF-κB and IRF3/7 activity downstream of PRRs in order to turn off and limit the production of type I IFNs.

## Discussion

The critical role that negative regulators play in TLR- and RLR-induced responses and in guarding against the onset of autoimmune and inflammatory diseases is demonstrated by the development of spontaneous disease in mice lacking these proteins such as TRIM21 and TIR8/SIGGIR [23], [37], [38]. Although at present there is broad recognition for the involvement of TRIMs in certain biological processes including cancer, retroviral restriction and autoimmunity [27], [39], [40], only recently has a real appreciation arisen for the specific and crucial role that TRIMs play as regulators of innate immune pathways [41], [42]. SPRY domain-containing TRIMs are thought to have multiple roles as anti-viral factors [43]. Whilst many of them, such as TRIM5α, have been shown to have direct anti-viral properties [44], others such as TRIM21, have been demonstrated to regulate type I IFN production in order to presumably prevent the overproduction of these potentially pathogenic cytokines [7], [8]. Thus, the negative effects of TRIM68 on IFN production observed are in keeping with this theory.

During this study we observed that TRIM68 is a potent negative regulator of type I IFN production, downstream of both TLR and RLR pathways. Results from reporter gene assays implied that TRIM68 was exerting its effect on type I IFN production downstream of TBK1. Proteomic analysis identified TFG as a *bona fide* TRIM68 target, a novel regulator of IFN-β induction, and hence an ideal target for TRIM68 in this respect. Most of the literature surrounding TFG demonstrates an involvement of this protein in cancer, with only two studies suggesting it plays a role in immune regulation [32], [45]. Regarding its role in cancer, TFG has been found to be preferentially up-regulated in prostate cancer cell lines and tissues, with TFG expression being associated with a higher probability of tumour recurrence following surgery [46]. In addition TFG has been identified as a putative metastatic melanoma tumour suppressor gene [47] and is also thought to play a role in non-small cell lung cancer, acting as a translocation partner for anaplastic lymphoma kinase (ALK) [48]. Miranda *et al* were the first to suggest TFG may play a role in immune regulation, showing that TFG over-expression can enhance the effect of TNF-α, TANK, TRAF2 and TRAF6 in inducing NF-κB activity suggesting that TFG is part of a complex containing TANK and IKK-γ [32]. We demonstrate for the first time that TFG can drive IFN-β promoter activity, an effect that is down-regulated in the presence of TRIM68, potentially via its effects on NF-κB as reported by Miranda *et al*. In fact, TBK1, although mostly appreciated for its role in IRF3 activation, has also been shown to play an important role in regulating NF-κB activity [49], [50]. Our results suggest that TRIM68 degrades TFG at a point of pathway convergence in which downstream events lead to the activation of both NF-κB and IRF3.

Recently it has become evident that a significant level of crosstalk exists between TRIF-driven and MyD88-driven pathways. For example TBK1 and IKKε have been shown to be activated by TLR ligands that signal via MyD88 and the canonical IKKs and the IKK-related kinases are thought to have several substrates in common in addition to their unique physiological substrates, such as IκBα and IRF3 [51], [52]. It is therefore possible that TFG may be part of the TBK1/IKKε/TANK/IKKγ signalosome complex, first identified by Chariot *et al* [53], and the degradation of TFG by TRIM68-mediated ubiquitination results in the disruption of this complex and subsequent inhibitory effect on type I IFN production. Although we could not establish the exact lysine residues involved in TRIM68 ubiquitination of TFG, it is possible that both K48 and K63 ubiquitin linkages play a part as both lysines have recently been shown to signal lysosomal degradation [54]. Interestingly, a recent study has suggested that TFG binds TRIM25 upon viral infection and negatively regulates RIG-I-mediated type I IFN signalling [45]. In contrast, we observed no inhibitory effects of TFG on IFN-β production, instead our results indicate a role for TFG in promoting IFN-β production.

Interestingly, both TRIM68 and TRIM21 are known autoantigens in SLE and primary Sjögrens syndrome (pSS), both autoimmune diseases associated with a high level of circulating type I IFN and an IFN-stimulated gene (ISG) signature [20], [31]. Whilst a number of recent studies have linked TRIM21 with the pathogenesis of SLE [23], [55], to date no known role for TRIM68 in regulating IFN production or potential involvement in SLE or pSS has been demonstrated [35], [56]. Interestingly however, a recent report has shown the *TRIM68* gene to be downregulated in pSS [57], potentially, given our results, resulting in a loss of negative regulation of type I IFN production and contributing to the enhanced levels of IFNs and ISG signature observed in pSS. An analysis of the role of TRIM68 and potentially TFG in pSS and SLE is therefore warranted.

In this study we demonstrate TRIM68 as a potent negative regulator of type I IFN production *via* degradation of the activatory protein TFG. We hypothesise therefore that a loss of TRIM68 activity or downregulation of expression may contribute to the overproduction of type I IFN typically observed in inflammatory conditions such as SLE and pSS. While the molecular pathways underpinning these inflammatory disorders are yet to be fully elucidated, modulation of TRIM68 activity may hold some potential as a novel therapeutic target for the treatment of disorders involving the overproduction of type I IFNs.

## Supporting Information

Figure S1 T-Coffee alignment of human TRIM68 and TRIM21. A clustal W alignment of TRIM68 and TRIM21 protein sequences.
(TIF)

Figure S2 TRIM68 negatively regulates NF-κB promoter activity. HEK293T cells were transfected with 40 ng full-length κB-luciferase reporter construct and 50 ng of the relevant promoter drivers MyD88, TRIF and TBK1 as well as with EV control and increasing amounts of TRIM68 as indicated. Cells were assayed for relative fold increase of reporter gene activity 18–24 hr post-transfection. Presented data is graphed from the average of three separate experiments ± S.E.M. *p<0.05 was considered significant.
(TIF)

Figure S3 TRIM68 potential interacting protein peptides were determined using Nano-LC MS/MS. A table of potential TRIM68 interacting proteins identified from excised gel fragment using MS/MS analysis. This list is compiled of peptide hits from two individual experiments. The “Global Proteome Machine (GPM)” http://www.thegpm.org/ and “Mascot” http://www.matrixscience.com/ home search engines were used to identify proteins from mass spectrometry data. The list includes protein name with swiss prot identifier (sp), the molecular weight of the protein in Daltons (Da), the pro peptide score, the cross correlation (XC) score and the sequence coverage percentage.
(TIF)

Figure S4 Lysines 48 and 63 are two possible lysines involved in TRIM68-mediated polyubiquitination of TFG. HEK293T cells were co-transfected with MYC-TFG, FLAG-TRIM68, wildtype HA-Ubiquitin, or ubiquitin mutants (HA-Ubiquitin K48R, HA-Ubiquitin K63R) for 18–24 hr. Ubiquitinated proteins were immunoprecipitated from lysates using anti-HA coated agarose beads and possible ubiquitination of TFG was assessed by immunoblotting with anti-MYC. Immunoblots shown are from a single experiment and are representative of two independent experiments.
(TIF)
