# Correction: TRIM68 Negatively Regulates IFN-β Production by Degrading TRK Fused Gene, a Novel Driver of IFN-β Downstream of Anti-Viral Detection Systems

**DOI:** 10.1371/journal.pone.0117957

**Published:** 2015-02-09

**Authors:** The PLOS ONE Staff

Dr. Angela Greco is not included in the author byline. She should be listed as the eighth author and affiliated with the Molecular Mechanisms Unit, Department of Experimental Oncology and Molecular Medicine, Fondazione IRCCS–Istituto Nazionale dei Tumouri, Milan, Italy. The contributions of this author are as follows: Contributed reagents/materials/analysis tools.
