# Supplementary figures and images for: TRIM68 Negatively Regulates IFN-β Production by Degrading TRK Fused Gene, a Novel Driver of IFN-β Downstream of Anti-Viral Detection Systems
Source: PLoS One. 2014 Jul 7;9(7):e101503. doi: 10.1371/journal.pone.0101503 (PMC4084880; doi:10.1371/journal.pone.0101503)

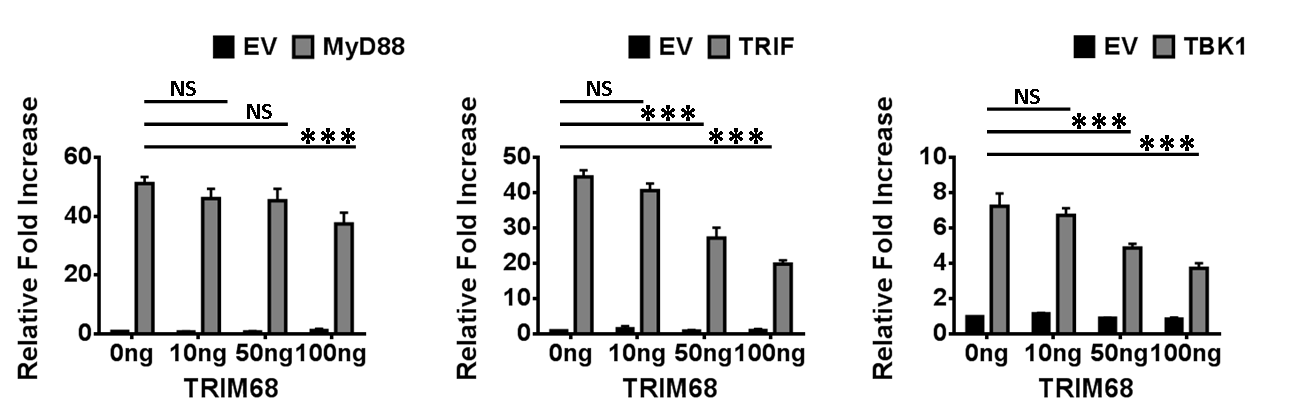

Supplement: Figure S2 — TRIM68 negatively regulates NF-κB promoter activity. HEK293T cells were transfected with 40 ng full-length κB-luciferase reporter construct and 50 ng of the relevant promoter drivers MyD88, TRIF and TBK1 as well as with EV control and increasing amounts of TRIM68 as indicated. Cells were assayed for relative fold increase of reporter gene activity 18–24 hr post-transfection. Presented data is graphed from the average of three separate experiments ± S.E.M. *p<0.05 was considered significant. (TIF) [file pone.0101503.s002.tif]

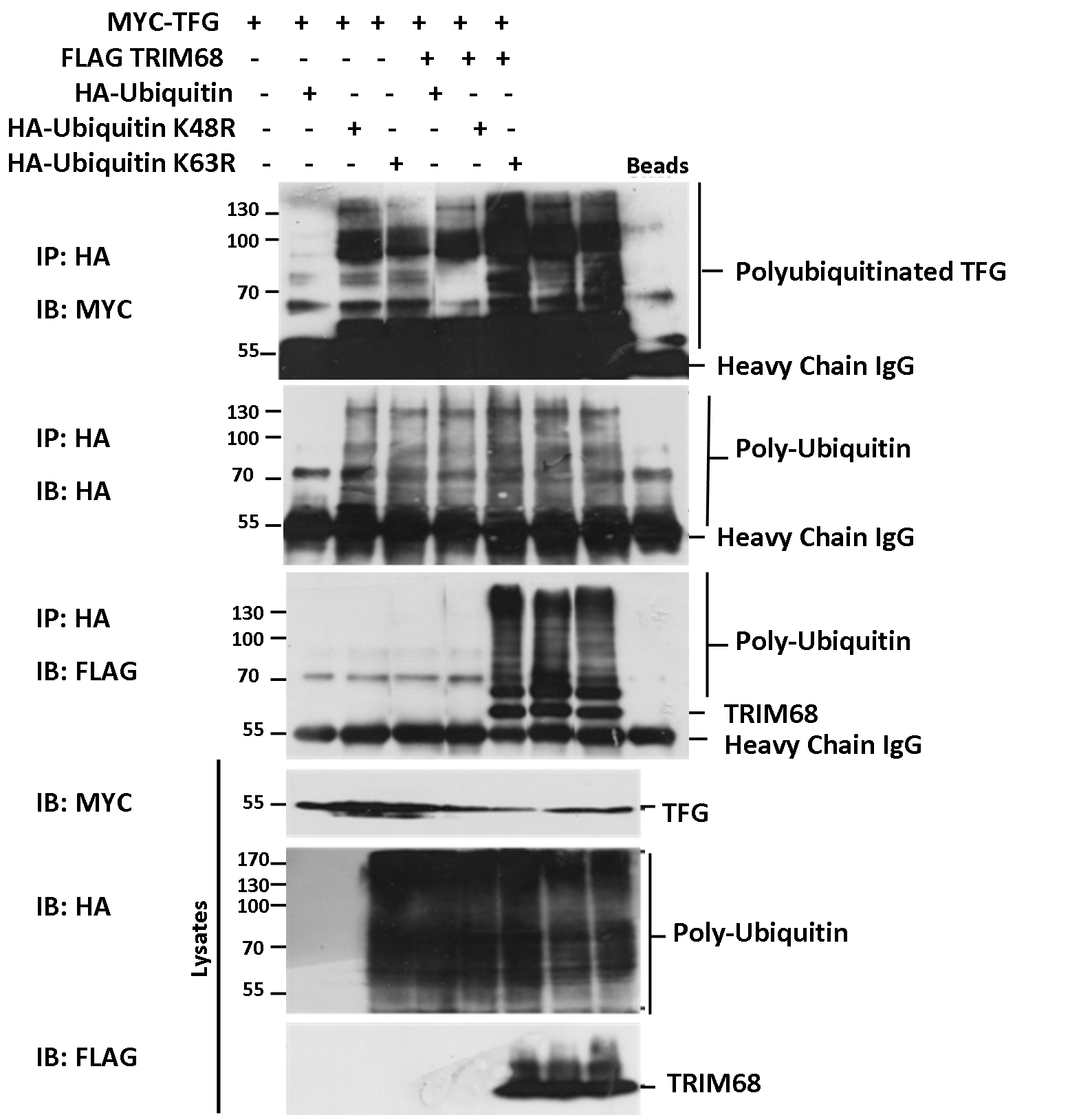

Supplement: Figure S4 — Lysines 48 and 63 are two possible lysines involved in TRIM68-mediated polyubiquitination of TFG. HEK293T cells were co-transfected with MYC-TFG, FLAG-TRIM68, wildtype HA-Ubiquitin, or ubiquitin mutants (HA-Ubiquitin K48R, HA-Ubiquitin K63R) for 18–24 hr. Ubiquitinated proteins were immunoprecipitated from lysates using anti-HA coated agarose beads and possible ubiquitination of TFG was assessed by immunoblotting with anti-MYC. Immunoblots shown are from a single experiment and are representative of two independent experiments. (TIF) [file pone.0101503.s004.tif]
